# *Acinetobacter baumannii* in the Age of Antimicrobial Resistance: Clinical Impact, Therapeutic Approaches, and Vaccine Development

**DOI:** 10.1155/bmri/7063234

**Published:** 2026-06-24

**Authors:** Krupa Bhaliya, Muneera Anwer, Hong Yin Wu, Quanlan Fu, Ming Q. Wei, Guoying Ni, Xiaosong Liu

**Affiliations:** ^1^ School of Pharmacy and Medical Science, Griffith University, Gold Coast Campus, Gold Coast, Queensland, Australia, griffith.edu.au; ^2^ The First Affiliated Hospital of Guangdong Pharmaceutical University, Guangzhou, China, gdpu.edu.cn; ^3^ Zhongao Biomedical Technology (Guangdong) Co. Ltd., Zhongshan, China

**Keywords:** *Acinetobacter baumannii*, antibiotic resistance, bacteriophage therapy, biofilm formation, multidrug resistance, outer membrane proteins, vaccine development, ventilator-associated pneumonia

## Abstract

*Acinetobacter baumannii* is a globally prevalent Gram‐negative pathogen responsible for a substantial proportion of healthcare‐associated infections, particularly in intensive care units. Its ability to persist in hospital environments, form biofilms on medical devices, and evade host immune responses has contributed to the rapid emergence of multidrug‐resistant (MDR) and extensively drug‐resistant (XDR) strains. This narrative review was conducted through a comprehensive literature search of PubMed, Scopus, Web of Science, and Google Scholar, focusing on studies published primarily over the past two decades. The review summarizes global epidemiology, vulnerable populations, mechanisms of antimicrobial resistance, clinical and economic impacts, and current and emerging therapeutic strategies. The advances in alternative treatments, including antimicrobial peptides, bacteriophage therapy, and vaccine development, are also discussed. In addition to summarizing existing knowledge, this review critically examines the key limitations underlying current therapeutic and vaccine strategies, including rapid resistance evolution, immune evasion, and antigenic variability. It further identifies major translational gaps and proposes future directions integrating systems biology and multitarget approaches. Although preclinical studies demonstrate the promising efficacy of several vaccine candidates, clinical trials in humans remain necessary to confirm their safety and effectiveness.

## 1. Introduction

*Acinetobacter baumannii* is a Gram‐negative, nonfermentative, rod‐shaped bacterium and a clinically significant pathogen, which is extensively studied within the field of medical and clinical microbiology. It is a typical member of the genus *Acinetobacter*, which comprises up to 47 recognized species [1]. *Acinetobacter* species are indicative of diverse ecological niches and have been found in the environment, skin, respiratory and digestive tracts, and other body sites. At present, only a few *Acinetobacter* species are known to cause disease, with *A. baumannii* being the most clinically relevant pathogen of the genus, exhibiting blood coagulation activity and binding to extracellular matrix and proteins such as laminin and fibronectin [2]. Disorders caused by *A. baumannii* range from mild clinical syndromes, such as bacterial pressure ulcers, pneumonia, urinary infections, and endocarditis, to sepsis, becoming significantly serious when infection is present in patients who use prolonged venous catheters, urinary catheters, intubation, and mechanical ventilation [3]. *A. baumannii* in healthcare causes significant harm to patients. Infection is typically fatal and tends to affect critically ill patients [4]. Ventilator‐associated pneumonia (VAP) and bloodstream infections (BSIs) are common, and mortality in ICU patients can reach 54%, so hospitals must be on high guard to prevent infection of vulnerable patients [5]. The capacity of *A. baumannii* to survive in healthcare and to evade treatment, means that an effective infection control must be strictly observed to protect patient safety. This organism accounts for one of the most important pathogens responsible for nosocomial infections (i.e., healthcare‐associated infections or HAIs), which include hospital‐acquired infections in hospitals, intensive care units (ICUs), and long‐term care facilities worldwide [6, 7]. It stands out as a particularly important pathogenic bacteria that can persistently survive for prolonged periods of time on different surfaces (a characteristic property that likely explains its transmission, or how bacteria move from one organism to another), in hospitals [8]. Indeed, hospital‐acquired infections with *A. baumannii* (named after a prominent pathologist) remain a serious healthcare issue, despite efforts to control its spread, in part due to the growing resistance of bacteria to antimicrobial drugs [9]. Aside from its medical relevance, it is a ubiquitous environmental soil/water isolate. *A. baumanni* contributes to the nosocomial infections, including its growing resistance to common and broad‐spectrum antibiotics, which makes its clinical management more difficult [10], is probably the best example of how important it is to medicine. *A. baumannii* contributes significantly to morbidity and mortality in hospitalized individuals due to its ability to survive for prolonged periods in nonliving environments, including indwelling devices. Combined with its tendency to be resistant to multiple classes of antibiotics, this ubiquitous and seemingly harmless commensal organism has become one of the most medically important and life threatening pathogenic bacteria of our time [11]. The spread of *A. baumannii*, including multidrug resistant (MDR) and extensively drug‐resistant (XDR) strains, in the United States, Europe, and China is also becoming a public health issue; carbapenem‐resistant *Acinetobacter baumannii* (CRAB), which emerges from the combination of the name carbapenem with *A. baumannii*, is particularly worrisome, given that this organism is often resistant to all or almost all active antibiotics. Thus, it is considered as one of the most challenging pathogens to treat [12]. *A. baumannii* is a notorious bacterium for its MDR ability and its prevalence in nosocomial (hospital‐acquired) infections. Unlike previous narrative reviews that primarily summarize epidemiology, resistance mechanisms, and therapeutic options, this review is aimed at providing a critical and integrative perspective on *A. baumannii* in the context of antimicrobial resistance (AMR). Specifically, this work links AMR, immune evasion, and therapeutic failure within a unified conceptual framework, highlighting key translational bottlenecks that limit the clinical success of both novel antibiotics and vaccine candidates. Furthermore, this review emphasizes emerging approaches, including systems biology, pan‐genomic analysis, and multiantigen vaccine strategies, as necessary directions to overcome current limitations. By shifting from a descriptive to a mechanistic and forward‐looking analysis, this review seeks to identify actionable gaps and inform future research and clinical translation.

## 2. Epidemiology and Vulnerable Populations

In the infections of *A. baumannii* are a common cause of healthcare‐associated infections. The pathogen is especially common in ICUs, where it accounts for about 2% of all healthcare‐associated infections, with most cases occurring in patients needing mechanical ventilation or those undergoing invasive procedures, according to the Centers for Disease Control and Prevention (CDC) [7]. The problem has been getting worse with CRAB infections, with as many as half of *A. baumannii* isolates being resistant to carbapenems [13]. According to the research in United States, a mortality rate of 26% in patients with CRAB infections were reported. In the United States, *A. baumannii* mainly targets critically ill patients in hospitals who are in ICUs and are on mechanical ventilation, central venous catheters or urinary catheters [14]. These patients are at the highest risk of getting VAP, BSIs and catheter‐associated urinary tract infections (CAUTIs) caused by *A. baumannii* [15]. Patients with diabetes, those needing surgeries, those who are on immunosuppressive drugs or who are immunocompromised, such as those with cancers, are at greater risk [16]. The CDC has listed *A. baumannii* as an organism of critical importance for patients staying in hospitals needing a long‐term care. *A. baumannii* is also a growing issue in Europe, particularly in southern and eastern European countries, where MDR and XDR strains have been responsible for significant outbreaks [17]. CRAB is endemic in many hospitals in southern Europe, in countries such as Greece, Italy, and Spain. According to the European Antimicrobial Resistance Surveillance Network (EARS‐Net), the prevalence of CRAB in ICUs in some southern European countries ranged from 50% to over 90% [18]. In southern European countries such as Italy and Greece, ICU outbreaks are particularly problematic in the setting of high rates of resistance and limited treatment options [12]. Outbreaks in these countries have also led to the spread of MDR *A. baumannii* to other European countries via transfers of patients. Like in the United States, the population at greatest risk of *A. baumannii* in Europe is the elderly in ICUs and long‐term care facilities. In European countries, such as Greece and Italy, where resistance is high, *A. baumannii* disproportionately affects elderly people as well as the immunocompromised. Patients on ventilators or with multiple comorbidities are at highest risk of developing an infection. There is some evidence that, in Italy, patients with prolonged hospitalization or high rates of prior antibiotic use are at greater risk [12]. In some parts of Europe, infections by *A. baumannii* have also been associated with trauma patients, such as those who were injured in a warzone. For example, there is a cluster of infections caused by *A. baumannii* affecting soldiers wounded in Iraq around 2004 and 2005 [19]. In China, *A. baumannii* has now come to be one of the most important nosocomial pathogens and there are frequent reports of MDR and XDR strains. MDR and XDR hospitals in major Chinese cities such as Beijing and Shanghai have been reporting increasing rates of CRAB infections, which further impede therapeutic options among critically ill patients. The frequency of MDR *A. baumannii* in China has been reported to be as high as 70% in some hospitals with a higher impact on respiratory infections [20]. The major population at risk in China is the one in ICUs, particularly patients on mechanical ventilation, who are more vulnerable to VAP. Older age and patients with a weakened immune system, or who are immunocompromised, with increased frequency in hospitals have high rates of MDR and CRAB infections. Neonates in NICUs are particularly vulnerable to infection, with several outbreaks reported in hospital settings of China [20].

CRAB has emerged as a major global health concern, with resistance rates exceeding 50% in several regions and reaching up to 90% in certain high‐burden settings. Recognizing its critical threat to human health, the World Health Organization has classified CRAB as a priority pathogen for the development of new antibiotics. These alarming trends highlight the urgent need for coordinated global surveillance and intervention strategies.

## 3. Economic Burden of *A. baumannii* Infections

*A. baumannii* infections contribute to a significant economic burden in hospitals where they are a common cause of infections. This is because infections of *A. baumannii* require long hospitalization and intensive care, and the cost of treating MDR infections is high. There are several factors attributed to significant economic burden of *A. baumannii* infections in hospitals. Firstly, patients with *A. baumannii* infections tend to stay in hospitals, often in ICUs, for longer periods of time, costing an estimated $25,000 to $35,000 per patient per day [10]. Treatment for these infections includes the administration of expensive, last‐resort antibiotics such as colistin or tigecycline or a combination of drugs to overcome resistance, as well as specialized medical therapies, such as the need for mechanical ventilation, dialysis, or extracorporeal membrane oxygenation (ECMO). Secondly, infections caused by *A. baumannii* have a high rate of treatment failure, patients often experience repeat infections or complications that require rehospitalization—increasing direct medical costs and placing an overall burden on the healthcare system through the needs for additional resources [21]. Lastly, the recovery time within *A. baumannii* infections is longer than among comparable infections, and the case fatality rates are higher, this results in a loss of productivity in the individuals, also affects their caregivers. In addition, in cases where the infection leads to chronic disability or death, there are downstream macroeconomic effects that include lost income and, ultimately, the burden this place on the families and communities where the afflicted individual lives [8]. In United States, the costs associated with *A. baumannii* infections are substantial. The CDC estimates the cost of treating CRAB infections to be more than $100,000 per patient; additional expenses accrue from longer hospital stays, more aggressive care in ICUs, and often expensive “last‐chance” antibiotics [22]. In Europe, the economic impact of *A. baumannii* is also significant. The European Centre for Disease Prevention and Control (ECDC) has stated that *A. baumannii* is one of main causes of hospital‐acquired infection in southern and eastern Europe [18]. In countries like Italy and Greece, the cost of treating *A. baumannii* infections is high; many hospitals report costing millions of euros. For example, spending on anti‐infection procedures and costly antibacterial medication rose. The indirect costs, such as loss of productivity and long‐term care, are added to the financial toll. In China, infections of *A. baumannii* are a major source of healthcare costs, particularly for those treated in tertiary hospitals. The overall cost to the healthcare system in China is difficult to put into dollar figures but has an estimated cost in the billions of yuan annually [20].

### 3.1. Impacts on Developing Countries

The worldwide burden of *A. baumannii* infections differs dramatically between developed and developing countries, and the nature of the infection is closely tied to vital aspects of healthcare infrastructure (including building construction, patient care and infection control), as well as antibiotic use. Due to the infection control measures (e.g., strict hygiene protocols, routine surveillance of HAIs, availability of advanced diagnostics), infections of *A. baumannii* are often well contained in the high‐resource settings of developed countries and, when they do occur, are recognized and treated. Hospitals in high‐income countries have the financial resources to isolate infected patients, execute strict antimicrobial stewardship programs, and utilize last‐line antibiotics appropriately [23]. In contrast, many low‐ and middle‐income countries struggle to maintain robust infection control standards because of meagre health infrastructure, crowded hospitals, and a lack of funding. Poor hygiene coupled with inadequate surveillance systems and limited access to diagnostic technologies increase infection rates resulting in a rise in *A. baumannii* infections [24]. For example, complicated infections caused by MDR *A. baumannii* in patients in rural Brazil led to a high mortality, a high readmission rate and prolonged hospitalization [25, 26]. Furthermore, exacerbated by antibiotic overuse and misuse, the emergence of MDR strains has increased rapidly in these regions [27]. In addition, treatment options in developed countries are more varied, because there are strong antibiotics and, if not, at least there is an option of bacteriophage therapy to treat *A. baumannii* [28]. Unfortunately, globally, and even in wealthy countries, the rise of these XDR and pan‐drug‐resistant (PDR) strains is a developing problem, because these strains are now resistant to almost all current antibiotics, and few new antibiotics are in development that might help. Treating them is becoming more and more difficult [25, 29]. The situation is still worse in the developing country, where access to the newer antibiotics is limited, older drugs remain in wide use, and treatment options are fewer. Given the prohibitive cost of last‐line antibiotics such as colistin, this makes the treatment in many healthcare facilities unaffordable, leaving patients defenseless against untreatable infections. This is compounded by poor access to healthcare, as well as the high availability of over‐the‐counter antibiotics. This is a major driver for the constant evolution of resistance and the continued dominance of nosocomial *A. baumannii* as a threat to public health [8]. Moreover, in high‐income countries, robust surveillance systems enable early recognition and control of *A. baumannii* outbreaks. In many low‐ and middle‐income countries, surveillance systems are incomplete, resulting in underreporting of *A. baumannii* infections with delays in control and the ongoing transmission of the pathogen, including the development of resistant strains [10, 30].

### 3.2. Major Organs and Tissues Affected by *A. baumannii* Infection

The most commonly affected organ in *A. baumannii* infections, in the United States, Europe, and China, is the lungs (usually in the form of VAP) [31]. The condition can have a high mortality when MDR strains of *A. baumannii* are involved [10]. Up to 40 per cent of VAP cases in ICUs in Europe can be attributed to *A. baumannii* infections [32]. BSIs also account for a significant proportion of *A. baumannii* infections identified in critically ill patients with central venous catheters. In the United States and Europe, BSIs are associated with a higher mortality, particularly in the context of patients infected with MDR and XDR strains [33]. CAUTIs are a second major complication of indwelling catheters in patients, which, thanks to biofilm formation, are difficult to eradicate in patients with healthcare‐associated infections around the United States and Europe [34]. In China, although *A. baumannii*‐induced CAUTIs are not as fatal as VAPIs, many such cases can be identified in high‐catheter‐use hospitals [35]. A common infecting agent for wounds of patients with burns or trauma, in the United States, it is a frequent cause of infections of war injuries in military personnel. The pathogen is also a common cause of infections in patients with traumatic injuries in Europe, where sometimes outbreaks in trauma and burn units have been reported [17, 32]—usually in hospitals with a higher rate of MDR *A. baumannii*. Central nervous system (CNS) infections of the brain (such as meningitis and brain abscesses) by *A. baumannii* are rare but potentially fatal and are seen in most patients with head trauma or after neurosurgical procedures. A few such cases have been reported in the United States, Europe, and China. Mortality in these cases can be high even with aggressive treatment [8, 35].

### 3.3. Pathogenesis of *A. baumannii* and Health Impacts

**Figure 1:**
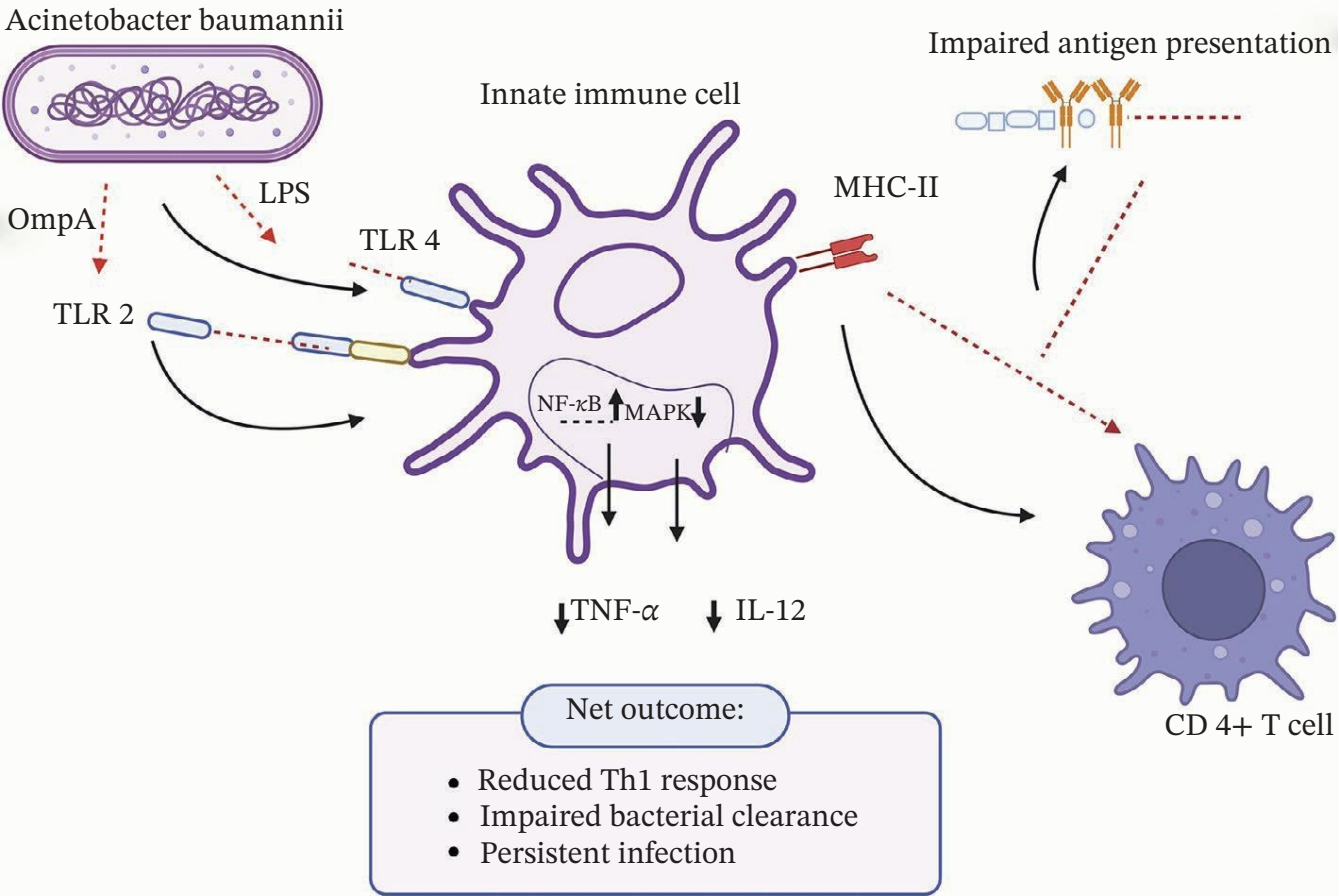
Immune evasion strategies employed by *Acinetobacter baumannii*.

*A. baumannii* is a complicated pathogen that exploits a range of molecular mechanisms to cause infections. It is a Gram‐negative rod‐shaped bacterium (occasionally appearing coccobacillary) that adapts efficiently to the host environments by using its small size to avoid immunity responses. The outer membrane proteins (OMPs) are one of the major molecular mechanisms exploited by this bug to escape the immune responses from the organism [8]. Altering its genetic content could also allow *A. baumannii* to develop antibiotic resistances and mechanisms of virulence, which will help it to survive in adverse locations [25]. *A. baumannii* is known to have a variety of virulence factors, such as characteristics that makes it more likely to cause diseases, including surface proteins that mediate adherence to host tissues and enzymes that break down host cellular structures. In addition, its ability to form biofilms makes it both easier to evade host immune responses, and better able to resist antibiotics [36]. Figure 1 illustrates the mechanisms used by *A. baumannii* to evade the innate immune response. Lipid A hepta‐acylation in LOS, capsular polysaccharides blocking complement and phagocyte activity, OmpA‐mediated complement inhibition and dendritic cell apoptosis, CipA and PKF protease interference with complement pathways, T2SS contribution to serum resistance, the Mla system limiting phospholipid exposure, degradation of neutrophil attractant phenylacetate, enhanced catalase activity against ROS, and intracellular survival/spread via neutrophils and macrophages. These virulence factors act together to take advantage of host vulnerabilities and cause significant infections. Genotypic and phenotypic diversity shown in analyses of *A. baumannii* isolates is high, being revealed in a study by Chen et al. [37, 38]. They emphasized a significant drug resistance against the carbapenem‐based antibiotic aztreonam, and the presence of the blaADC‐25 gene primarily on chromosome contigs. They noted overall seven sequence types, indicating a robust level of genetic heterogeneity. Multilocus sequence typing, as well as plasmid grouping, become critical tools for controlling infection and tailoring therapy [39]. *A. baumannii*′*s* proclivity for biofilm formation is one of major factors in its tenacity in clinical settings. Biofilms protect the bacteria to survive on medical equipment, surfaces, and even develop a resistance to antibiotics [40]. Biofilm formation is discussed by Francesca Longo in 2024 as, biofilms can make infections worse and more difficult to treat. In addition to providing a physical layer of protection, the bacterium′s embrace of biofilms augments bacterial tolerance enabling *A. baumannii* to survive harsh conditions including hot temperatures and active chemical disinfectants. Factors that contribute to the organism′s renowned ability to withstand many disinfectants and antibiotics used in hospitals. The formation of biofilm plays a major role in the pathogenesis of *A. baumannii* [41]. It protects bacterial cells from the host immune responses and antibiotic treatment. Approximately all *A. baumannii* isolates can form biofilms. Biofilms contribute to persistent infections particularly in implanted medical [42]. Chronic infection might be a possible result of these persisting biofilms and complicate the courses of therapies. *A. baumannii* is increasingly highlighted as an important public health threat due to its remarkable adaptability and notorious ability to resist antibiotics as a highly antibiotic‐resistant bacterium. It does so via multiple mechanisms, including beta‐lactamase production or upregulation; decreased membrane permeability; modification of aminoglycoside or tetracycline target sites; and antibiotic efflux [43]. There has been an alarming escalation in numbers of infections by these strains, which are resistant to the most recently elected last‐resort antibiotics, namely to the polymyxin colistin, tigecycline and most recently the carbapenems, and their rapid spread in the community. *A. baumannii* is most notorious for its MDR, which is due to the activities of several efflux pumps that are responsible for pumping antibiotics out of its cells; this bacterium develops different resistance mechanisms, including altering antibiotic target sites and producing enzymes that inactivate the drug effect [44]. Genetic plasticity endows *A. baumannii* the ability to gain resistance genes at an astounding rate, often rendering infections untreatable and resulting in enhanced morbidity and mortality. Although we are now better at treating infections of *A. baumannii*, clinically it is striking how the bacteria continue to overtake the development of new drugs. Its resistance strategies will continue to be a target for a therapy and a challenge for medicine until we fully understand them and are able to treat infections with both new antibiotics and more refined approaches to manage resistant infections. This is complicated by the growing frequency of MDR *A. baumannii*, and thus therapies such as phage therapy or vaccines are needed to control outbreaks or infection prevention [45]. Here are some major pathogeneses caused by *A. baumannii.*

Importantly, AMR in *A. baumannii* is not an isolated phenomenon but is closely interconnected with virulence and immune evasion. Biofilm formation, for example, not only enhances resistance to antibiotics but also protects the bacteria from host immune responses. Similarly, modifications in outer membrane structures contribute both to reduced drug permeability and immune escape. This interconnected “resistance–virulence–immune evasion axis” represents a critical conceptual framework for understanding the persistence and clinical impact of *A. baumannii* infections. Targeting this integrated network, rather than individual mechanisms, may provide more effective therapeutic and preventive strategies.

#### 3.3.1. VAP

*A. baumannii* is an important cause of VAP, mainly in critical care patients. VAP involves the colonization of the respiratory tract by *A. baumannii,* followed by invasion of the lungs of critically ill patients on mechanical ventilation and the development of serious respiratory distress, with very high mortality. This condition is often very hard to treat owing to the intrinsic MDR of the pathogen [10, 30].

#### 3.3.2. BSIs

BSIs caused by *A. baumannii* are associated with high rates of morbidity and mortality, especially in patients with an indwelling catheter in a central vein (called an indwelling central venous catheter) or who are undergoing other invasive procedures. Once in the bloodstream, it can trigger sepsis, a systemic inflammatory response, and a life‐threatening condition that causes organ failure. The mortality rate for patients with sepsis caused by *A. baumannii* is much higher than for sepsis caused by other bacterial pathogens, often because it is MDR [8, 46].

#### 3.3.3. CAUTIs

*A. baumannii* is a frequent cause of CAUTIs, including in hospitalized patients with chronic or long‐term use of catheters. The bacteria can attach to and form biofilms on the surfaces of catheters, resulting in recalcitrant or chronic infections that are relatively difficult to treat. If CAUTIs are left untreated, they can develop to more severe complications, such as pyelonephritis or urosepsis [34, 47].

#### 3.3.4. Wound Infections

The ability of *A. baumannii* to generate/form biofilms in wound tissues helps it to persist and hide from antimicrobials, ultimately leading to chronic infections. The ability of *A. baumannii* to cause difficult‐to‐treat wound infections has become evident in war zones and disaster settings, leading to one of its many nicknames: “Iraqibacter” (after Iraq because of its high prevalence in hotspots of military conflict). Many wounded soldiers and civilians have developed severely infected wounds, often with limited access to antibiotics and surgical care [48]. Often, these cases can be mistaken for gangrene, which further complicates the treatment. *A. baumannii* also made headlines during the Bhopal factory explosion in 1984 [49], where a lethal combination of methyl isocyanate gas, water, and residual pesticides sloshed down the walls of a factory in central India, intoxicated thousands, and left hundreds of people with severely burned skin [49].

#### 3.3.5. Meningitis

Although uncommon, *A. baumannii* can cause meningitis in patients with head trauma or neurosurgical procedures. *A. baumannii* meningitis is often associated with poor outcomes due to the organism′s antibiotic resistance and poor pharmacokinetics of the drugs in the cerebrospinal fluid (CSF) [8, 29].

### 3.4. Antibiotic Resistance Mechanisms

*A. baumannii* is one of the biggest challenges of modern medicine due to its high potential for antibiotic resistance. It employs several different mechanisms to resist antibiotics, making infections very hard to control. These mechanisms include degrading enzymes, modification of antibiotic targets, efflux pumps, and outer‐membrane modifications. Among all, the most characterized are the beta‐lactamases, which hydrolyze the beta‐lactam ring in antibiotic molecules, rendering them inactive. Of particular concern are the OXA‐type carbapenemases (e.g., OXA‐23, OXA‐24, and OXA‐58) that confer carbapenem resistance, a class of drugs often used as a last line against infection. Recent work has shown how these carbapenemase genes have spread widely among clinical isolates, making the treatment of MDR infections difficult [50, 51]. Besides, efflux pumps, such as AdeABC, actively “pump” drugs such as antibiotics out of the bacterial cell, reducing the concentrations and efficacies of the antibiotics inside the bacteria. These efflux pumps belong to the resistance‐nodulation‐division (RND) family and can impart resistance to multiple common antibiotic classes, not just one, including beta‐lactams, aminoglycosides, and fluoroquinolones. Recent work [52] has shown that the formation of efflux pumps, specifically efflux pumps that are overexpressed, is often highly correlated with the formation of biofilm. Another important mechanism of AMR involves modifications to the outer membrane that hinder the passage of antibiotics into the bacterial cell. The downregulation or mutation of porins—which are global protein channels embedded within the outer membrane allowing the passage of a diversity of antibiotics—often decreases the efficacy of drugs, including carbapenems [53]. Other modifications to lipopolysaccharides (LPS)—the high‐molecular‐weight compounds that form the core of the outer leaflet in the outer membrane of all bacterial species—also contribute to resistance against polymyxins, such as colistin, which is often the last antibiotic‐class used to treat MDR‐AB infections [54]. Horizontal gene transfer also speeds up the dissemination of resistance genes between bacterial strains. *A. baumannii* acquires resistance genes from plasmids, transposons, and integrins, enabling the rapid spread of MDR and carbapenem‐resistant strains even in a healthcare environment. Gene transfer can be further facilitated via biofilms whereby the close neighborhood of bacterial cells might also enhance the exchange of genetic material [55, 56]. Additionally, *A. baumannii* can also undergo adaptive resistance, a phenotypic mechanism by which the bacteria generate new characteristics during its lifespan, such as the process of phase variations and persister cells. Persister cells are a small subpopulation of bacteria that are dormant and survive an antibiotic therapy then emerge after the antibiotic pressure has been released. These adaptive mechanisms are more often seen in bacteria associated with biofilms and make *A. baumannii* infections chronic [57].

### 3.5. Treatments for Infections of *A. baumannii*

Themanagement of *A. baumannii*Infections are particularly challenging because this pathogen can evolve multiple resistance mechanisms, including MDR, XDR, and, in some instances, PDR phenotypes [58]. The treatment of A. baumannii infections is challenging due to its high antibiotic resistance. Standard antibiotics that used to be effective, like beta‐lactams (including penicillins and cephalosporins) and aminoglycosides, are often ineffective. Currently available treatment strategies are listed in Table 1. Antimicrobial peptides (AMPs) are small proteins with antimicrobial activities, produced by various organisms including our body. Bacteriophage therapies using DNA sequencing technology, pharmaceutical companies are now rapidly isolating and characterizing phages responsible for lysing *A. baumannii* bacterial cells. Using computational methods, the development of synthetic phage has allowed us to design “wild‐type killer” viruses [59, 60]. This approach enhances the abilities of these viruses to recognize and kill strains lacking virulence.

**Table 1:** Currently available antibiotics against *A. baumannii,* along with their resistance mechanisms.

Antibiotic class	Representative agents	Current resistance status	Key resistance mechanisms	References
**Penicillins**	Ampicillin and piperacillin	High resistance	Intrinsic resistance and *β*‐lactamases	[10]
***β*-lactam/*β*-lactamase inhibitor combinations**	Piperacillin–tazobactam and ampicillin–sulbactam	Variable	OXA‐type enzymes; sulbactam intrinsic activity	([61]; [10])
**Cephalosporins (all generations)**	Ceftriaxone, ceftazidime, and cefepime	Near‐universal resistance	AmpC cephalosporinases and OXA‐type *β*‐lactamases	[62]
**Carbapenems**	Imipenem and Meropenem	High resistance	OXA‐23, OXA‐24/40 and OXA‐58 carbapenemases	[63]
**Monobactams**	Aztreonam	Ineffective	*β*‐lactamases and poor permeability	[62]
**Aminoglycosides**	Amikacin and gentamicin	Variable	Aminoglycoside‐modifying enzymes and efflux	[10]
**Fluoroquinolones**	Ciprofloxacin and levofloxacin	High resistance	GyrA/parC mutations and efflux pumps	[62]
**Tetracyclines**	Doxycycline and minocycline	Moderate activity	Tet genes and efflux systems	[37, 38]
**Glycylcyclines**	Tigecycline	Limited/salvage therapy	AdeABC efflux pump overexpression	[37, 38]
**Polymyxins**	Colistin and polymyxin B	Limited effectiveness	Lipid A modification (pmr genes)	[64]
**Rifamycins**	Rifampicin	Combination use only	Rapid resistance when used alone	[65]
**Folate pathway inhibitors**	Trimethoprim–sulfamethoxazole	High resistance	Target mutations	[62]
**Novel/siderophore cephalosporins**	Cefiderocol	Emerging resistance	*β*‐lactamases and iron transport alterations	[66]

AMR, including MDR, signifies a major and measurable global health issue affecting humans, animals, and the environment, highlighting its identification as a significant One Health concern. Global estimates suggest that bacterial AMR was linked to around 4.7–5 million deaths in 2019, with 1.1–1.3 million directly due to drug‐resistant infections, underscoring a significant impact on human health comparable to numerous leading causes of death globally [67]. Additionally, the extensive use of antibiotics in livestock production increases the selection and distribution of resistant bacteria; numerous studies demonstrate the direct transmission of resistant pathogens from animals to humans through food, physical contact, and environmental pathways [68]. Environmental reservoirs worsen this issue by spreading antibiotic residues and resistant traits into soil and water ecosystems, promoting the horizontal transfer of resistance genes among bacteria across various sectors [69]. Due to the convergence of resistance in these areas, there has been a rapid global increase in MDR infections. Recent studies have shown increases in significant hospital‐related and community‐acquired resistant infections, and projections indicate that AMR‐related deaths could rise significantly in the absence of coordinated intervention [70]. Together, these findings demonstrate that AMR/MDR is not exclusive to healthcare settings but rather represents an expanding, interrelated problem in people, animals, and the environment that must be assessed and addressed with all‐encompassing One Health monitoring and intervention techniques.

### 3.6. Current Antibiotic Therapies

#### 3.6.1. Carbapenems

Carbapenems are a class of antibiotics including imipenem, meropenem, and doripenem—used to be the mainstay of treatment for *A. baumannii* but CRAB strains, promoted by carbapenem‐hydrolyzing enzymes such as OXA‐type beta‐lactamases, are increasingly common. Research involving whole‐genome sequencing identified new markers for CRAB strains [51, 71]. These researchers observed that genes encoding carbapenemases in *A. baumannii* have spread globally. Carbapenems are often used as the last resort for treating MDR strains of *A. baumannii*. However, carbapenem‐resistant strains have emerged, which limits their effectiveness.

#### 3.6.2. Polymyxins

Colistin (polymyxin E) and polymyxin B are the last‐line treatments for infections caused by MDR *A. baumannii*, but they are nephrotoxic and neurotoxic and have been widely reported to induce colistin‐resistant strains. Recent findings from a study in Nature Communications [61, 72] demonstrate the multiple mechanisms by which *Acinetobacter* can become resistant to colistin, involving lipid A modification and alterations to the outer membranes. Polymixins are usually used in combination therapy for carbapenem‐resistant strains; however, nephrotoxicity is a major concern and requires a new treatment option.

#### 3.6.3. Tigecycline

Tigecycline has been shown to be active against MDR *A. baumannii*; however, its suboptimal pharmacokinetics (pharmacokinetics are the ways that a drug behaves in your body, especially in patients with BSIs) limits the clinical utility of this compound. Additionally, in the recent reviews published in Nature Microbiology [73, 74], novel tigecycline resistance mutations were demonstrated in *A. baumannii*, further emphasizing the urgency of new therapies.

### 3.7. Emerging Antibiotics

#### 3.7.1. Cefiderocol

A novel siderophore cephalosporin called cefiderocol has also demonstrated strong activities against CRAB, a major pathogen found in VAP, by actively targeting the bacterial iron uptake system, while circumventing bacterial resistance mechanisms to effectively inhibit bacterial cell wall synthesis [75]. A paper in Nature Reviews Drug Discovery asserts cefiderocol as “the first novel gram‐negative antimicrobial agent in over 20 years,” with an exceptional activity against Gram‐negative pathogens including VAP and BSIs [76].

#### 3.7.2. Plazomicin

As a new‐generation aminoglycoside, plazomicin is resistant to many aminoglycoside‐modifying enzymes and has been effective against MDR pathogens. In a recent study, the activity of plazomicin against *A. baumannii* was highlighted, with particular focus on overcoming classic resistance to aminoglycosides [77].

#### 3.7.3. Eravacycline

Importantly, the fluorocycline antibiotic eravacycline has shown activities against MDR Gram‐negative bacteria, including *A. baumannii*, but its pharmacokinetics raise doubts regarding its activities when used against BSIs. Articles in Nature Reviews Drug Discovery emphasized that eravacycline can be considered as an active option for complicated intra‐abdominal infections due to MDR pathogens [78, 79].

### 3.8. Why Do New Antibiotics Rapidly Fail?

Although several novel antibiotics, including cefiderocol, eravacycline, and plazomicin, have demonstrated activity against MDR *A. baumannii*, their clinical effectiveness is increasingly challenged by the rapid emergence of resistance. One key factor is the remarkable genetic plasticity of *A. baumannii*, which enables rapid acquisition and dissemination of resistance determinants. Mutations affecting iron transport systems, particularly those associated with cefiderocol uptake, have already been reported, reducing drug efficacy.

Additionally, the overexpression of efflux pumps and alterations in membrane permeability further limit intracellular antibiotic concentrations. These mechanisms often act synergistically, conferring resistance across multiple drug classes. Pharmacokinetic and pharmacodynamics limitations also contribute to therapeutic failure. For example, suboptimal tissue penetration and reduced efficacy in BSIs limit the clinical utility of certain agents such as tigecycline. Importantly, the rapid emergence of resistance even to newly developed antibiotics highlights that antimicrobial innovation alone is insufficient. A more integrated strategy targeting resistance mechanisms, bacterial persistence, and host–pathogen interactions is required to achieve sustained therapeutic success.

### 3.9. Development of AMPs

One potential therapeutic strategy that can overcome MDR *A. baumannii* is the application of AMPs. These are endogenously produced proteins by the body that kill bacteria by interacting with their outer membranes, leading to rapid lysis of the cells. Since they target the cell membranes of bacteria (the cellular envelope), AMPs are not susceptible to the common resistance mechanisms. Bacteria are less likely to develop resistance against them because they would not need to change their membranes [80]. As a rule, AMPs work by targeting the bacterial membrane, interacting with the negatively charged LPS responsible for destabilizing membranes and lysis of the cells. A recent review in Nature Reviews Microbiology examined the prospects of AMPs as therapeutic weapons to combat MDR pathogens, due to their broad‐spectrum activity and their potential use against *A. baumannii* [81]. LL‐37 is a human cathelicidin‐AMP that has shown promising activities against *A. baumannii*. Ongoing work on compounds designed to optimize potency and stability is presented in a review paper published in Nature Communications [71]. For polymyxin derivatives, it is stated in the journal of Nature Microbiology that modifying polymyxins to limit nephrotoxicity while retaining antimicrobial efficacy is especially important in the face of colistin‐resistant strains of *A. baumannii* [82]. Cationic antimicrobial peptides (CAMPs) such as defensins and magainins are being considered as an option for treating *A. baumannii* infections, as they destabilize bacterial membranes to cause death [83].

### 3.10. Bacteriophage Therapy

Phage therapy is based on the use of bacteriophages (phages), viruses that infect and kill bacteria, including MDR bacteria. The potential therapeutic benefits include an important advantage that phage is extremely selective and does not harm the normal flora in the human body [84] so the rest of the bacterial species in the host remain unharmed. Phage preparations have been used as antimicrobials for humans for 100 years. Currently, the phage therapy potential of *A. baumannii* as an antibacterial agent is being studied in vivo [85]. The efficacy of phage preparation against carbapenem‐resistant and XDR *A. baumannii* has been investigated [86]. In addition, adjunct therapies incorporating AMPs or bacteriophages as supplements to antibiotics also show promise to enhance treatment efficacy. One recent report showed that combination therapies comprising colistin paired with rifampin or tigecycline substantially enhanced the in vitro outcomes against colistin‐resistant *A*. *baumannii* [61].

### 3.11. Vaccine Development

With the increasing resistance to antibiotics and the persistence of *A. baumannii* in hospital settings, developing a vaccine has become an important goal. Vaccine development against *A. baumannii* has been underway and targets common antigens, such as outer membrane porins and capsular polysaccharides. While referencing to strain variability has complicated the development of an overarching, pan‐strain vaccine, several promising candidates are moving forward through preclinical stages, and hopefully, preventative measures could be put in place [87]. A few vaccine candidates have been designed for *A. baumannii*, but their clinical efficacies have only been tested in a few mouse models. The vaccine candidates mainly include whole‐cell inactivation, polysaccharides, or outer membrane vesicles (OMVs). The generated immune responses are mainly mediated by antibodies, and the mice vaccinated with the inactivated bacteria or OMVs exhibited protection from intraperitoneal lethal challenge with *A. baumannii* cells [88]. Additionally, the project is aiming to exploit structure‐based methods and synthetic chemistry for the development of a cross‐DNA and antigen‐based vaccine that can target all Gram‐negative pathogens, including *A. baumannii*; further studies are highly necessary for the development of an effective vaccine candidate targeting *A. baumannii* [89]. Unlike other bacterial infections, natural immunity to *A. baumannii* is typically weak and short‐lived, making reinfections common. The failure of natural infection to confer long‐term immunity has been recognized, which has reinforced the need for vaccination as a means of inducing robust and durable immunity [90]. Innate immune responses on neutrophils and macrophages, whereas adaptive responses to *A. baumannii* infection involve humoral (antibody) responses against surface antigens. Particularly noteworthy is the ability of the bacterium to modulate various host‐signaling pathways that might otherwise allow for appropriate immune clearance [56]. Despite the diversity of vaccine platforms explored, each approach faces distinct translational limitations, as summarized in Table 2.

**Table 2:** Vaccine platforms and key translational limitations.

Platform	Advantage	Key limitation
OMP‐based	Strong immunogenicity	High strain variability
OMVs	Broad antigen exposure	Reactogenicity concerns
CPS	Targets virulence	High heterogeneity
Whole‐cell	Strong immune response	Safety concerns
mRNA/DNA	Flexible design	No bacterial success yet

### 3.12. Current Vaccine Strategies Under Development

#### 3.12.1. Subunit Vaccines

Subunit vaccines (so called because they contain an antigenic subunit of the relevant pathogenic bacterium) use specific bacterial proteins or polysaccharides to spur the immune response. This approach is safer than the live‐attenuated vaccines.

##### 3.12.1.1. OMPs

**Figure 2:**
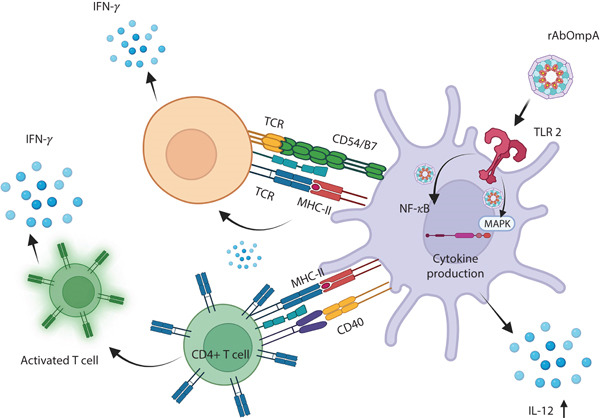
Immune modulation by outer membrane vesicles of *Acinetobacter baumannii*.

OMPs (particularly OmpA and OmpW) are identified as major virulence factors responsible for mucin binding and host cell evasion, playing a significant role in *A. baumannii* being a MDR pathogen. Preclinical studies have demonstrated that OmpA‐based vaccines can induce both humoral and cellular immune responses and have provided partial protection against *A. baumannii* in animal models. Encouragingly, recent studies suggest that OmpA‐directed vaccines could attenuate bacterial colonization and biofilm formation. For example, in a study published in 2018, mice were vaccinated with OmpA conjugated with an aluminum adjuvant, followed by a challenge with a lethal dose of *A. baumannii* [91]. They found that the vaccine reduced the bacterial load and significantly rescued the survival of the challenged mice. Furthermore, subsequent studies have shown that OmpA‐targeted vaccines reduce bacterial colonization and biofilm formation. In a study by Hassan et al. [92] published in 2021, the authors combined OmpA‐1 and OmpA‐2 (which together constitute more than 20% of the OMPs composition). Their study demonstrated that this approach reduces bacterial colonization and biofilm formation. Figure 2 shows immune modulation by *A. baumannii* OMVs and OMV‐associated AbOmpA, where AbOmpA binds to pattern recognition receptors on antigen‐presenting cells, leading to upregulation of MHC‐II and costimulatory molecules, activation of CD4+ T cells, and subsequent stimulation of B cells to produce OMV‐specific antibodies, accompanied by proinflammatory cytokine release.

##### 3.12.1.2. Biofilm‐Associated Proteins (Bap)

Bap is a key component of biofilm formation, which allows *A. baumannii* to cling to medical devices. Indeed, vaccines targeting Bap prevented biofilm formation and improved bacterial clearance by the immune systems in mouse models and in a preclinical model of pneumonia (pneumonic bacterial infection), as previously described in Journal of Bacteriology [93]. These findings suggest that vaccines inducing antibodies to Bap could improve immune recognition and killing of *A. baumannii*.

##### 3.12.1.3. Capsular Polysaccharides

The polysaccharide capsule of *A. baumannii* itself confers immune evasion by inhibiting phagocytosis, so immune evasion is another promising antivirulence target, and this could have vaccine potential. In fact, a recent review in Nature Reviews Microbiology even highlighted the potential of vaccination against capsular polysaccharides as a vaccine strategy, “given their role in virulence and host immune resistance” and their diagnosability, although their heterogeneity would decrease its appeal for the development of universal vaccines [56].

### 3.13. Whole‐Cell and Inactivated Vaccines

#### 3.13.1. Inactivated Whole‐Cell (IWC) and Bacterial Ghost (BG) Vaccines

Research has explored IWC vaccines and BGs as potential vaccine candidates for future [94]. Animal models using inactivated vaccines have demonstrated reduced bacterial burden in the lungs, spleen, and other organs after immunization [36], and it was recently reported that an inactivated *A. baumannii* vaccine could protect against lethal *A. baumannii* infection in a murine animal model, although safety and potential reactogenicity in humans are still issues [92].

### 3.14. DNA Vaccines

The next strategy to be discussed is DNA vaccines, a new approach to combating *A. baumannii*. DNA vaccines code bacterial antigens carried in plasmids that are injected into the host, where they express the antigens and subsequently stimulate the host′s immune responses [95]

#### 3.14.1. Plasmid‐Based DNA Vaccines

Additional plasmids encoding other antigens, such as OmpA and Bap, have been tested in animal models and found to induce both humoral immune responses and cell‐mediated immune responses. Recent improvements in the stability and effectiveness of DNA vaccine technology mean that these could eventually enter clinical use [92].

### 3.15. mRNA Vaccines as an Emerging Approach

mRNA vaccines represent a new field of study for *A. baumannii*. After the rapid development of mRNA vaccines for COVID‐19, emerging research has focused on the development of mRNA vaccines for bacterial pathogens, such as *A. baumannii*. mRNA vaccines provide several advantages including speedy development, flexibility, and the potential to induce both humoral and cellular immunity against a pathogen [96].

1. Rapid development: mRNA vaccines can be designed to target key virulence factors or conserved antigens in *A. baumannii* and could be rapidly modified to reflect the composition of emerging strains, including those with an MDR phenotype [36].
2. Targeting virulence factors: mRNA vaccines encoding antigens such as OMPs or Bap that are fundamental to the virulence of *A. baumannii*, is an interesting approach to prevent bacterial colonization and to stop the formation of biofilm [92].
3. Barriers: Making mRNA vaccines work for bacteria is more difficult due to the unique structure of their cells and their abilities to evade the immune system through various mechanisms such as biofilm and capsule formation [97]. However, if vaccine strategies manage to overcome these barriers, they can be more effective because the bacteria must be directly targeted through specific antigens of the bacterial cells.

Although, *A. baumannii*‐specific mRNA vaccines are in the early stages of research, and nasal and inhaled mRNA vaccines have not yet been developed, they could 1 day prevent infections with MDR strains [98].

### 3.16. Progresses in Animal Models

Animal models are essential to test the efficacy and safety of *A. baumannii* vaccines before advancing to the clinical use. Murine models remain the most common, but other animal models such as rabbits and nonhuman primates are being developed to study vaccine candidates before testing in humans to extrapolate which one might expect from the vaccine in human trials. Over the past few years, a few vaccine candidates have shown encouraging results in animal studies.

#### 3.16.1. Murine models

Mouse models are commonly used to test new *A. baumannii* vaccines, as they are more readily available than other organisms and are known to reproduce closely similar infection processes to humans. In an evaluation of the protective efficacy of a vaccine based on the capsular polysaccharide, it was demonstrated that mice immunized with this molecule had significantly lower bacterial counts in the lung and spleen after infection with *A. baumannii* [99]. Meanwhile, in studies using an OmpA‐based vaccine, Singh et al. [91] found a protective immune response that resulted in a significant reduction in bacterial colonization [91].

#### 3.16.2. Pneumonia Models

Animal models of *A. baumannii*‐induced pneumonia are also being used to evaluate vaccine candidates, developing inactivated and live bacterial vaccines for protection against the onset of VAP, a complication associated with intensive care admissions [36]. A whole‐cell inactivated vaccine candidate has been proven protective in murine pneumonia, reducing mortality rates and improving bacterial clearance, as recently reported.

#### 3.16.3. Biofilm‐Related Studies in Animal Models

Considering the significance of biofilm formation in *A. baumannii* pathogenesis, animal models have been used to test vaccines against biofilm‐associated proteins. Biofilm‐associated proteins, including Bap, have been shown to be targets of vaccination that can prevent chronic infection by reducing biofilm formation. Such vaccines might work nicely for the prevention of infections associated with implanted medical devices, where biofilm formation plays a significant role in the pathogenesis of infection [93].

#### 3.16.4. Sepsis and Systemic Infection Models

Animal models of sepsis in mice can be used to test vaccines that target virulence factors. Immunization with OMV vaccines in mice reduced *A. baumannii* dissemination and increased the survival rates in models of *A. baumannii*‐induced sepsis [100].

### 3.17. Why Has No *A. baumannii* Vaccine Reached Clinical Use?

Despite extensive preclinical progress, no vaccine against *A. baumannii* has successfully advanced to clinical use. This translational gap reflects several fundamental biological and immunological challenges.

First, natural immunity to *A. baumannii* is typically weak and short‐lived, limiting the ability to generate durable protective responses following infection. Reinfections are common, suggesting that the pathogen does not induce strong immunological memory.

Second, significant antigenic heterogeneity across strains undermines the effectiveness of single‐antigen vaccine strategies. Surface‐exposed targets such as OMPs and capsular polysaccharides exhibit considerable variability, reducing cross‐protection.

Third, immune evasion mechanisms—including biofilm formation, capsule‐mediated shielding, and modulation of host immune responses limit antigen accessibility and impair effective immune recognition.

Finally, the absence of well‐defined immune correlates of protection remains a major barrier to vaccine development. Without clear markers of protective immunity, it is difficult to rationally design and evaluate vaccine candidates.

Collectively, these challenges suggest that future vaccine strategies must move beyond single‐target approaches toward multiantigen, systems‐level designs that account for pathogen diversity and immune complexity. As highlighted in Table 2, these limitations collectively contribute to the lack of clinical translation.

## 4. Conclusion

*A. baumannii* is a microbe that poses an enormous threat to global healthcare as it is highly antibiotic‐resistant, persistent in hospital environments, and causes infections in many critically ill patients, particularly those patients in ICUs on mechanical ventilators or with indwelling medical devices. It places a large economic burden on our healthcare system with an increased hospital length of stay and healthcare costs, particularly for MDR and XDR strains.

Although last‐line antibiotics such as colistin and novel therapies such as bacteriophage treatment might find some success in treating infections, the problem of evolving resistance also serves as a reminder that new antibiotics and effective vaccines are still urgently needed. Work on AMPs and vaccines is underway, but there is still much that needs to be done to help control the spread of this dangerous pathogen in resource‐poor countries. A successful vaccine would be a great game‐changer in the way that *A. baumannii* infections would be managed and prevented globally. Current treatments are limited, and new antimicrobial agents and therapies are in development to address this growing threat. Additionally, vaccine development is still in the early stages and requires more extensive research to be carried out before testing in clinical settings. As this pathogen continues to evolve, research needs to address both immediate clinical concerns and long‐term solutions. Future progress will depend on integrated strategies targeting the resistance–virulence–immune evasion axis rather than isolated therapeutic approaches.

### 4.1. Future Directions

*A. baumannii* remains a challenging pathogen, especially in hospital settings, due to its virulence, ability to form biofilms, and, most critically, its antibiotic resistance. Given the global rise in MDR bacteria, continued research into both treatment and prevention options for *A. baumannii* is crucial. Although there are several vaccine candidates that work well in animal models, none has yet been tested in humans. The move from preclinical to clinical prototypes will likely require intensive safety and efficacy reviews in the most vulnerable segments of the population. Adding adjuvants might be an important addition to improve vaccine′s efficacy against the bacterium *A. baumannii*, according to a review in Nature Reviews Drug Discovery by Carlos Vaara [83]. Exploring the efficacy of emerging adjuvants might be considerable for future vaccine development [101]. Furthermore, when combined with a drug such as antibiotics or phage therapy, vaccination may prove to be particularly protective or additive. Another promising avenue is the use of probiotics as adjunctive therapy. By modulating the gut and respiratory microbiota, producing antimicrobial metabolites, and enhancing host immunity, specific probiotic strains may reduce colonization by MDR pathogens [102, 103]. Although evidence remains preliminary and strain‐specific, probiotics could be utilized to represent a cost‐effective preventive strategy in high‐risk settings such as ICUs and warrant further investigation. The future of *A. baumannii* research will require multidisciplinary approaches combining immunology, microbiology, epidemiology, and pharmacology. Efforts will be needed across multiple fronts, including antibiotic development, vaccine research, infection control, and diagnostic innovation. Given the global threat posed by this pathogen, continued investment in research is essential to improve patient outcomes and curb the spread of this highly resistant and virulent bacterium.

## Author Contributions

Krupa Bhaliya: writing—original draft and final revisions. Muneera Anwer: writing—original draft, review and editing, software, proofreading, and final revisions. Hong Yin Wu: review. Quanlan Fu: review. Ming Q. Wei: review. Guoying Ni: review. Xiaosong Liu: conceptualization.

## Funding

Open access publishing facilitated by Griffith University, as part of the Wiley–Griffith University agreement via the Council of Australasian University Librarians.

## Conflicts of Interest

The authors declare no conflicts of interest.

## Data Availability

Data sharing is not applicable to this article as no datasets were generated or analyzed during the current study.
